# Interference with Ca^2+^-Dependent Proteolysis Does Not Alter the Course of Muscle Wasting in Experimental Cancer Cachexia

**DOI:** 10.3389/fphys.2017.00213

**Published:** 2017-04-19

**Authors:** Fabrizio Pin, Valerio G. Minero, Fabio Penna, Maurizio Muscaritoli, Roberta De Tullio, Francesco M. Baccino, Paola Costelli

**Affiliations:** ^1^Department of Clinical and Biological Sciences, University of TurinTurin, Italy; ^2^Department of Clinical Medicine, Sapienza UniversityRome, Italy; ^3^Department of Experimental Medicine, University of GenovaGenova, Italy

**Keywords:** muscle protein turnover, calpains, calpastatin, muscle atrophy, proteostasis

## Abstract

Protein hypercatabolism significantly contributes to the onset and progression of muscle wasting in cancer cachexia. In this regard, a major role is played by the ATP-ubiquitin-proteasome-dependent pathway and by autophagy. However, little is known about the relevance of the Ca^2+^-dependent proteolytic system. Since previous results suggested that this pathway is activated in the skeletal muscle of tumor hosts, the present study was aimed to investigate whether inhibition of Ca^2+^-dependent proteases (calpains) may improve cancer-induced muscle wasting. Two experimental models of cancer cachexia were used, namely the AH-130 Yoshida hepatoma and the C26 colon carcinoma. The Ca^2+^-dependent proteolytic system was inhibited by treating the animals with dantrolene or by overexpressing in the muscle calpastatin, the physiologic inhibitor of Ca^2+^-dependent proteases. The results confirm that calpain-1 is overexpressed and calpastatin is reduced in the muscle of rats implanted with the AH-130 hepatoma, and show for the first time that the Ca^2+^-dependent proteolytic system is overactivated also in the C26-bearing mice. Yet, administration of dantrolene, an inhibitor of the Ca^2+^-dependent proteases, did not modify tumor-induced body weight loss and muscle wasting in the AH-130 hosts. Dantrolene was also unable to reduce the enhancement of protein degradation rates occurring in rats bearing the AH-130 hepatoma. Similarly, overexpression of calpastatin in the tibialis muscle of the C26 hosts did not improve muscle wasting at all. These observations suggest that inhibiting a single proteolytic system is not a good strategy to contrast cancer-induced muscle wasting. In this regard, a more general and integrated approach aimed at targeting the catabolic stimuli rather than the proteolytic activity of a single pathway would likely be the most appropriate therapeutic intervention.

## Introduction

Cachexia is a wasting syndrome that frequently occurs in cancer patients, worsening both their prognosis and quality of life and significantly reducing survival rate. Although at different degrees, virtually all body compartments are affected by cachexia, the skeletal muscle being among the most compromized. Previous studies showed that cancer-induced muscle wasting mainly derives from acceleration of protein breakdown rates. The main proteolytic systems operating in the muscle were all found hyperactivated in tumor hosts, suggesting that they could be considered as potential therapeutic targets (reviewed in Argilés et al., 2014). Recent reports however, showed that inhibition of proteasome-dependent proteolysis or autophagy was not able to improve muscle wasting in tumor-bearing animals (Penna et al., 2013, 2016).

The contribution of proteasomes and autophagy to the onset and progression of cancer-induced muscle wasting was deeply investigated, however quite little is known about the Ca^2+^-dependent proteolytic system.

Calpains (EC 3.4.22.17) are a family of Ca^2+^-dependent cysteine proteases. Three members of this family are mainly expressed in the skeletal muscle, namely the ubiquitous μ- and m-calpains and the muscle-specific p94 calpain (also known as calpains-1, -2, and -3). Both μ- and m- calpains consist of two subunits of 80 and 30 kDa, respectively (reviewed in Ono et al., 2016). The former (constituted by four domains) contains the catalytic cleft, whereas the latter (two domains) is endowed with regulatory functions. The p94 calpain also includes three insertions that are not shared with other calpains. When intracellular Ca^2+^ concentrations increase, inactive calpains, normally localized in the cytosolic compartment, translocate to the cell membrane and the 80 kDa subunit is converted by autoproteolysis to a 75 kDa form. A number of cytoskeletal proteins were proposed as substrates of calpains, among which desmin, dystrophin, fodrin, myosin, troponins I, and T (reviewed in Ono et al., 2016). Other reports showed that also calpastatin, the physiologic inhibitor of calpains, and the plasmamembrane 130 kDa Ca^2+^-ATPase are cleaved by Ca^2+^-dependent proteases (Pontremoli et al., 1991; Salamino et al., 1992; Ono et al., 2016).

The physiological relevance of calpains is demonstrated by their involvement in several processes such as cell proliferation, differentiation, migration, apoptosis, and aging. Ca^2+^-dependent proteases were also proposed to contribute to different pathologies. A cause-effect relationship was established among calpain deficiencies and diseases, for this reason defined calpainopathies (Ono et al., 2016). As for cancer cachexia, increased calpain mRNA levels were reported in the skeletal muscle of animals bearing the AH-130 hepatoma (Busquets et al., 2000). The contribution of calpain activity to muscle wasting induced by tumor growth in rats bearing the Yoshida AH-130 hepatoma was suggested by the progressive reduction of both calpastatin and the 130 kDa Ca^2+^-ATPase levels (Costelli et al., 2001), as well as by the increased cleavage *in vitro* of specific fluorogenic substrates (Costelli et al., 2002; Borges et al., 2014). More recently increased calpain expression was reported in the muscle of tumor-bearing rats treated with sorafenib (Toledo et al., 2016). In patients with cancer, however, both increased or unchanged muscle calpain levels were observed (Smith et al., 2011; Tardif et al., 2013). Differencies in tumor type and disease severity likely account for such discrepancies.

Although a number of data support the involvement of Ca^2+^-dependent proteolysis in cancer cachexia, protein hypercatabolism was not suppressed in muscles isolated from tumor-bearing animals and exposed to calpain inhibitors (Temparis et al., 1994; Baracos et al., 1995; Llovera et al., 1995). Such studies, however, were performed on preparations of isolated muscles incubated in the presence of calpain inhibitors, which is a rather non-physiological setting. Previous studies from our laboratory showed that leupeptin, an inhibitor of cysteine proteases including calpains and many lysosomal cathepsins, improved muscle wasting in rats bearing the AH-130 hepatoma (Tessitore et al., 1994).

On the whole, while some evidences do not support a role of calpain system in cancer-associated muscle wasting, other findings suggest the opposite. Thus, the working hypothesis in the present study is that specific inhibition of calpain activity could be a means to prevent, or at least delay, muscle wasting that occurs in cancer cachexia. Along this line, the calpain system was modulated in tumor-bearing animals either by pharmacological or genetic means.

## Materials and methods

### Reagents

All reagents supplied by Sigma-Aldrich (St. Louis, MO, USA), unless differently specified.

### Animals and treatments

Male Wistar rats (5 weeks old/150 g) or male Balb/c mice (5 weeks old/20 g) were provided by Charles River, Calco, Italy. They were housed on a regular dark-light cycle (light from 08:00 to 20:00), with free access to food and water, and cared for in compliance with the Italian Ministry of Health Guidelines and the Policy on Humane Care and Use of Laboratory Animals (NIH, 1996). The experimental protocol was approved by the Bioethical Committee of the University of Turin. Both rats and mice were randomized into two groups, namely controls and tumor hosts. As for rats, (*n* = 8) they were injected intraperitoneally (i.p.) with 10^8^ AH-130 Yoshida ascites hepatoma cells, whereas tumor-bearing mice (*n* = 6) were subcutaneously (s.c.) inoculated between the shoulder blades with 5 × 10^5^ Colon 26 (C26) carcinoma cells. Control animals (rats: *n* = 8; mice: *n* = 6) received saline, i.p. or s.c. In a second experiment rats were divided into four groups (*n* = 8): Controls, dantrolene treated controls, AH-130 hosts and dantrolene-treated AH-130 hosts. Dantrolene (10 mg/kg b.w.) was daily administered s.c., starting on the day of tumor transplantation. Finally, another experiment using the C26 tumor was performed. the animals were divided into two groups, namely controls (C) and tumor hosts (C26). Each group was further divided into transfected (*n* = 6 for both C and C26) and untransfected (*n* = 6 for both C and C26). Transfection procedure is described below.

AH-130 hosts and C26-bearing mice were euthanized under isoflurane anesthesia. The former were sacrificed at days 4 or 7 after tumor transplantation, while sacrifice time for the latter was day 14 of tumor growth. Several muscles and organs were rapidly excised, weighed, frozen in isopentane cooled with liquid N_2_, and stored at −80°C for subsequent analysis.

### Histological analysis

Serial 10 μm-thick frozen sections were cut from cryopreserved tissue blocks, adhered to Superfrost Plus microscopy slides and stained with hematoxylin and eosin. All sections were examined by light microscopy (Nikon Eclipse TS100) and digital images were obtained with a Nikon COOLPIX 4500 camera. To assess myofiber cross sectional area (CSA) ~300–400 fibers of *tibialis anterior* muscle sections were counted and measured using the Image J software (http://rsb.info.nih.gov/ij/; NIH, Bethesda, MD).

For immunofluorescence, transverse sections were fixed in 4% paraformaldehyde and probed with the primary anti-calpastatin mAb 35,23 (1:200 Melloni et al., 2006). Detection was performed using a FITC-conjugated mouse IgG secondary antibody (1:2,000; Bio-Rad Laboratories, Hercules, CA). Nuclei were stained with the DAPI fluorochrome and the images captured with a Nikon COOLPIX 4500 camera in an epiilluminated fluorescence microscope (Axiovert 35, Zeiss, Germany).

### Protein turnover

Muscle protein turnover rates were determined as previously described (Costelli et al., 1993). Briefly, rats received an i.p. dose of NaH^14^CO3 (250 μCi/kg b.w.) 24 h before tumor transplantation. They were then sacrificed at days 0 and 7 after tumor inoculation and total protein radioactivity determined. Briefly, gastrocnemius was rapidly weighed and homogenized to 10% (wt/vol) in chilled water. Total protein content was determined by the method of Lowry et al. (1951) using bovine serum albumin as working standard. Trichloroacetic acid-insoluble proteins were processed for lipid extraction, extensively hydrolyzed, and counted in a liquid scintillation spectrometer to obtain total protein radioactivity. Fractional rates of protein degradation (k_d_) were calculated as follows and expressed as %/day:

$$\begin{array}{l} {\text{k}}_{\text{d}}=\text{ln}(\text{total protein radioactivity})/\text{t}. \end{array}$$

### Calpastatin transfection

RNCAST600 cloned in pEYFP-C1 (De Tullio et al., 2009) was used to express a calpastatin form containing the regulatory L-domain and a single inhibitory unit. The left *tibialis anterior* muscle was injected with 25 μl of 0.5 U/μl hyaluronidase (to improve transfection efficiency) and 2 h later injected with 50 μg of plasmidic DNA, while the contralateral muscle served as control. One minute following DNA injection transcutaneous pulses were applied by two stainless steel plate electrodes (gap between plates: 4 mm). Electrical contact with the leg skin was assured by shaving each leg and applying conductive gel. Electric pulses with a standard square wave were delivered by an electroporator (ECM-830, BTX-Harvard Apparatus, Holliston, MA, USA). Three pulses (20 ms each) of 75 V/cm were administered to the muscle with a delivery rate of 1 pulse/s. The polarity was then reversed and a further three pulses were delivered to the muscle. Electroporation was performed 10 days before animal sacrifice. With the transfection procedure described, no sign of muscle damage and inflammatory infiltrate could be seen by histological analysis (Penna et al., 2010). The expression of YFP-RNCAST600 was confirmed by western blotting (see below), using an anti-GFP antibody (Figure S1; Santa Cruz Biotechnology, Santa Cruz, CA, USA).

### Western blotting

Approximately 50 mg of gastrocnemius or 25 mg of tibialis muscle were homogenized in 80 mmol/L Tris-HCl, pH 6.8, containing 100 mmol/L dithiothreitol, 70 mmol/L SDS, and 1 mmol/L glycerol, with freshly added protease and phosphatase inhibitor cocktails; kept on ice for 30 min; centrifuged at 15,000 × g for 10 min at 4°C and the supernatant collected. Protein concentration was assayed according to Bradford, using bovine serum albumin as working standard. Equal amounts of protein (30 μg) were heat-denaturated in sample-loading buffer (50 mmol/L Tris-HCl, pH 6.8, 100 mmol/L dithiothreitol, 2% SDS, 0.1% bromophenol blue, 10% glycerol), resolved by SDS-PAGE, and transferred to nitrocellulose membranes (Bio-Rad Laboratories, Hercules, CA). The filters were blocked with Tris-buffered saline containing 0.05% Tween and 5% non-fat dry milk and then were incubated overnight with a mouse monoclonal anti-calpain-1 antibody (1:1000; Chemicon, Temecula, CA, USA, Milan, Italy), recognizing the catalytic subunit (~80 kDa) of calpain-1; a commercial anti-calpastatin antibody recognizing the whole protein (1:200; Santa Cruz Biotechnology, Santa Cruz, CA, USA) and the monoclonal anti-calpastatin antibody mAb 35,23 recognizing also the partially digested forms of calpastatin (1:1,000; provided by R. De Tullio, characterization described in Melloni et al., 2006); an antibody against tubulin (1:8,000, mouse monoclonal antibody, clone T5168); or GAPDH (1: 5,000, goat polyclonal antibody, Santa Cruz Biotechnology, Santa Cruz, CA). Peroxidase-conjugated IgGs (Bio-Rad Laboratories, Hercules, CA) were used as secondary antibody. For quantification, performed by densitometric analysis (TotalLab; Non-linear Dynamics, Newcastle on Tyne, UK), samples were run on a single gel (representative patterns shown in Figures) and normalized against tubulin or GAPDH levels.

### Data analysis and presentation

Results are expressed as means ± *SD*. The significance of the differences was evaluated by analysis of variance (ANOVA) followed by Tukey's test or by Student's “*t*”-test (indicated in Figure legend). As for the fractional rates of protein turnover, the significance of the differences was calculated by comparing linear regression by ANOVA (Lee and Lee, 1982).

## Results

Rats bearing the Yoshida AH-130 hepatoma and mice implanted with the C26 colon carcinoma show a marked loss of both body weight and muscle mass (Costelli et al., 1993; Bonetto et al., 2009).

**Figure 1:**
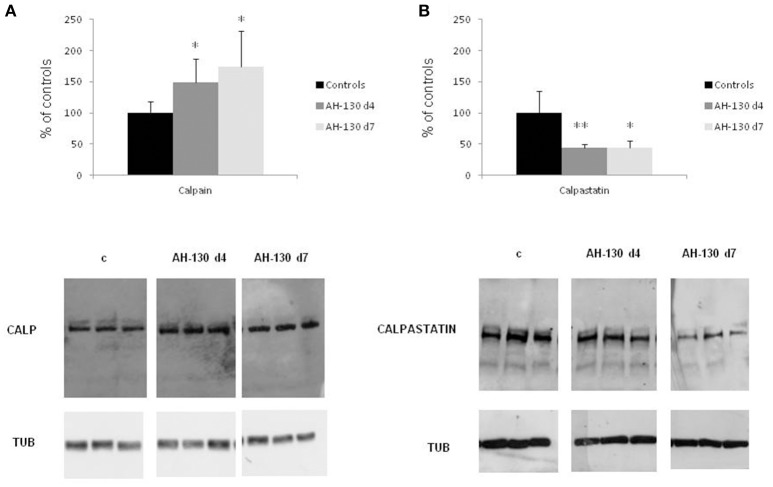
**Calpain and calpastatin expression in controls and rats bearing the AH-130 hepatoma. (A)** Calpain protein expression in tibialis muscles of controls and AH-130 tumor-bearing rats after 4 and 7 days from tumor inoculation. **(B)** Calpastatin protein expression in tibialis muscles of controls and AH-130 tumor-bearing rats after 4 and 7 days from tumor inoculation. Densitometric quantifications (histograms) were normalized according to tubulin levels. Blot representative patterns are shown. Data (mean ± *SD, n* = 8) are expressed as % of controls. Significance of the differences (ANOVA): ^*^p < 0.05; ^**^p < 0.01 vs. controls.

**Figure 2:**
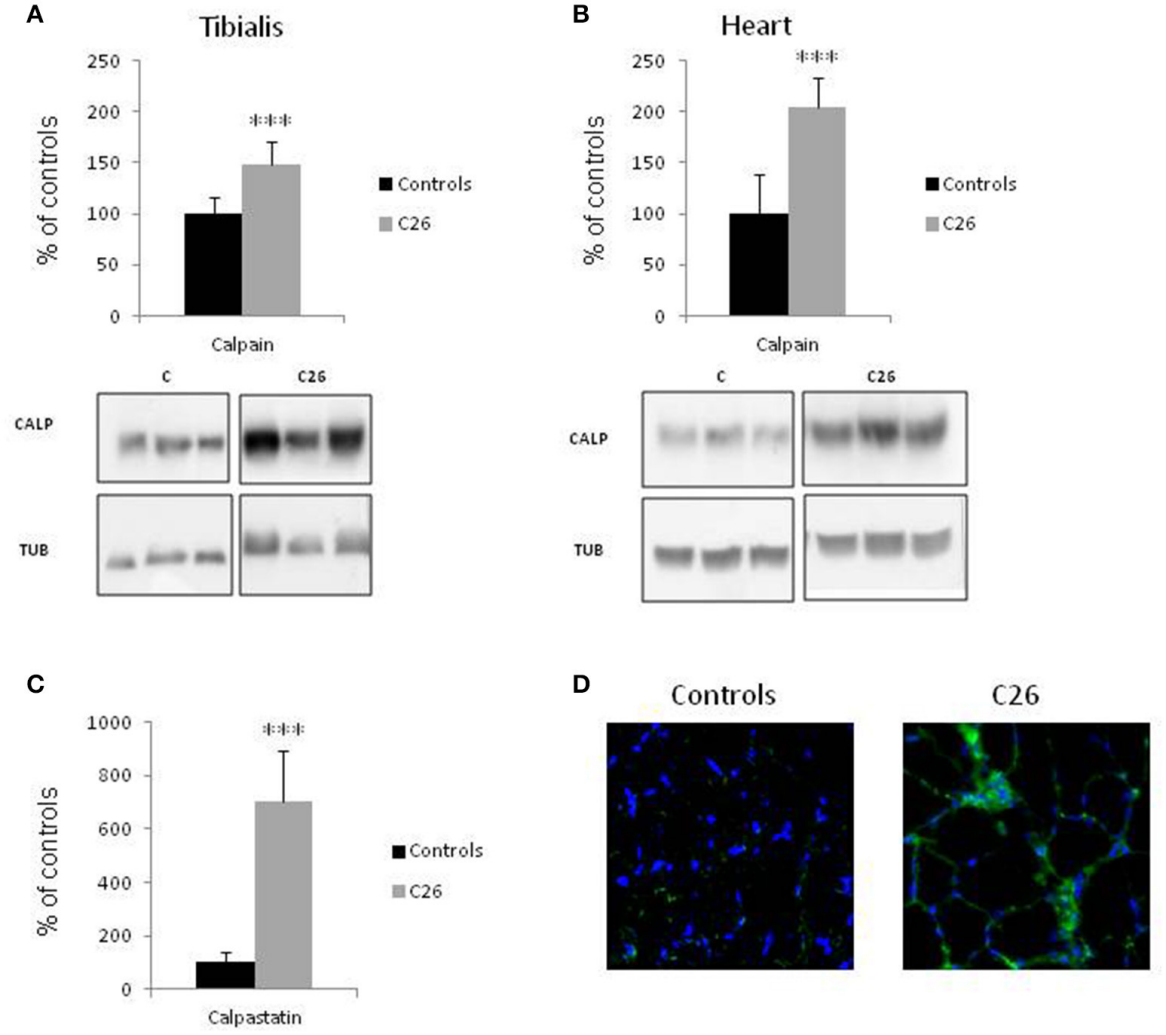
**Calpain and calpastatin expression in control animals and in mice implanted with the C26 tumor**. Calpain protein levels in the tibialis muscle **(A)** and in the heart **(B)** of controls and C26 hosts. **(C)** Calpastatin digestion products were detected in the tibialis muscle of controls and C26 tumor-bearing mice by the mAb 35,23. **(D)** Immunofluorescence images of tibialis muscle sections in controls and C26-bearing mice stained for calpastatin (green) and nuclei (blue). Densitometric quantifications (histograms) were normalized according to tubulin levels. Blot representative patterns are shown. Data (mean ± *SD, n* = 6) are expressed as % of controls. Significance of the differences (*t*-test): ^***^p < 0.001 *vs*. controls.

Skeletal muscle wasting is associated with enhanced activity of the Ca^2+^-dependent proteolytic system. In the muscle of rats bearing the AH-130 hepatoma, the levels of calpain-1 are increased while those of the endogenous specific inhibitor calpastatin are reduced (Figures 1A,B; Costelli et al., 2001). Similarly, calpain-1 levels are increased in both the *tibialis anterior* (Figure 2A) and heart (Figure 2B) of mice implanted with the C26 tumor. In parallel, digestion products of calpastatin, markers of calpain activity (De Tullio et al., 2000), accumulate in the muscle of C26 hosts (Figure 2C and Figure S1). Finally, in these latter calpastatin is diffused in the cytosol (Figure 2D), a localization that is associated with calpain activation and Ca^2+^ increase (De Tullio et al., 1999).

**Figure 3:**
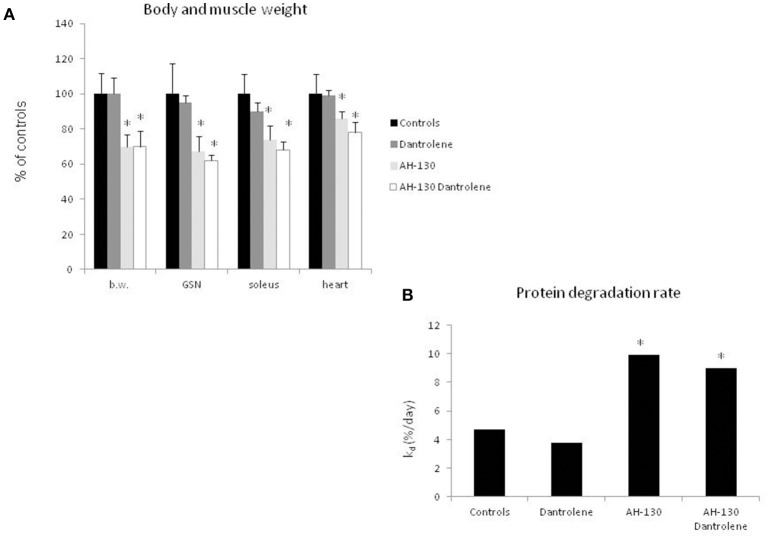
**Effects on treatment with dantrolene on muscle weight and protein degradation rates in rats bearing the AH-130 hepatoma. (A)** Body weight (b.w.), gastrocnemius (GSN), soleus, and heart weight in controls and AH-130 tumor-bearing rats. The data are reported as % of controls (mean ± *SD, n* = 8). Significance of the differences (ANOVA): ^*^p < 0.05 vs. controls. **(B)** Fractional rates of protein degradation in the gastrocnemius muscle, expressed as % day (mean ± *SD, n* = 8), in controls and AH-130 hosts, treated or not with dantrolene. Significance of the differences (linear regression comparison; see Materials and Methods): ^*^p < 0.05 vs. controls.

To understand if interfering with the activity of the Ca^2+^-dependent proteolytic system can antagonize cancer-induced muscle wasting, rats bearing the AH-130 hepatoma were treated with dantrolene, a molecule that inhibits Ca^2+^ release from the sarcoplasmic reticulum, thus reducing calpain activation (Perry et al., 2010; Malvestio et al., 2016). The results show that dantrolene administration did not prevent either the loss of body and muscle weight (Figure 3A and Table S1) or the acceleration of protein breakdown rates (Figure 3B). No appreciable effects of dantrolene could be observed on tumor mass (total cell number: 1,198 × 10^6^ ± 239 and 1,480 × 10^6^ ± 359 in untreated and treated AH-130 hosts, respectively) and on food intake (C = 136 ± 17 g, C + dantrolene = 129 ± 22 g, AH-130 = 94 ± 10 g, AH-130 + dantrolene = 85 ± 16 g, *p* < 0.01 tumor hosts vs. controls, irrespective of treatment). In addition, dantrolene did not exert any effect on control rats (Figure 3). Similar data were obtained using a cell-permeable calpain inhibitor (Figure S2).

**Figure 4:**
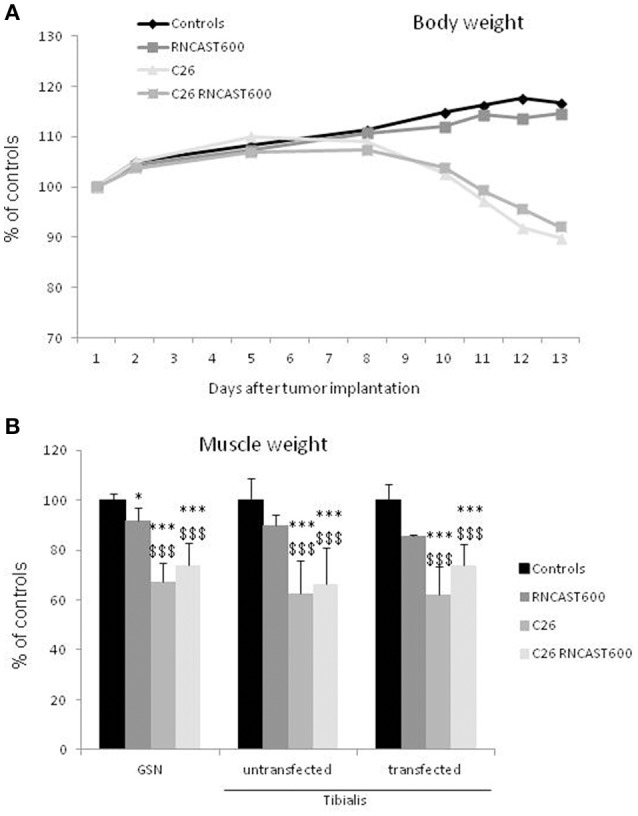
**Calpastatin overexpression in the tibialis muscle of controls and C26 hosts. (A)** Body weight of controls and C26-bearing mice transfected (RNCAST600) and untransfected. The data are reported as % of controls (means ± *SD*; d_0_ C = 18.72 ± 0.373 g). **(B)** Gastrocnemius (GSN) and tibialis muscle weight. Data are expressed as % of controls (mean ± *SD, n* = 8). Significance of the differences (ANOVA): ^*^p < 0.05; ^***^p < 0.001 vs. controls, ^$$$^*p* < 0.001 vs. RNCAST600.

**Figure 5:**
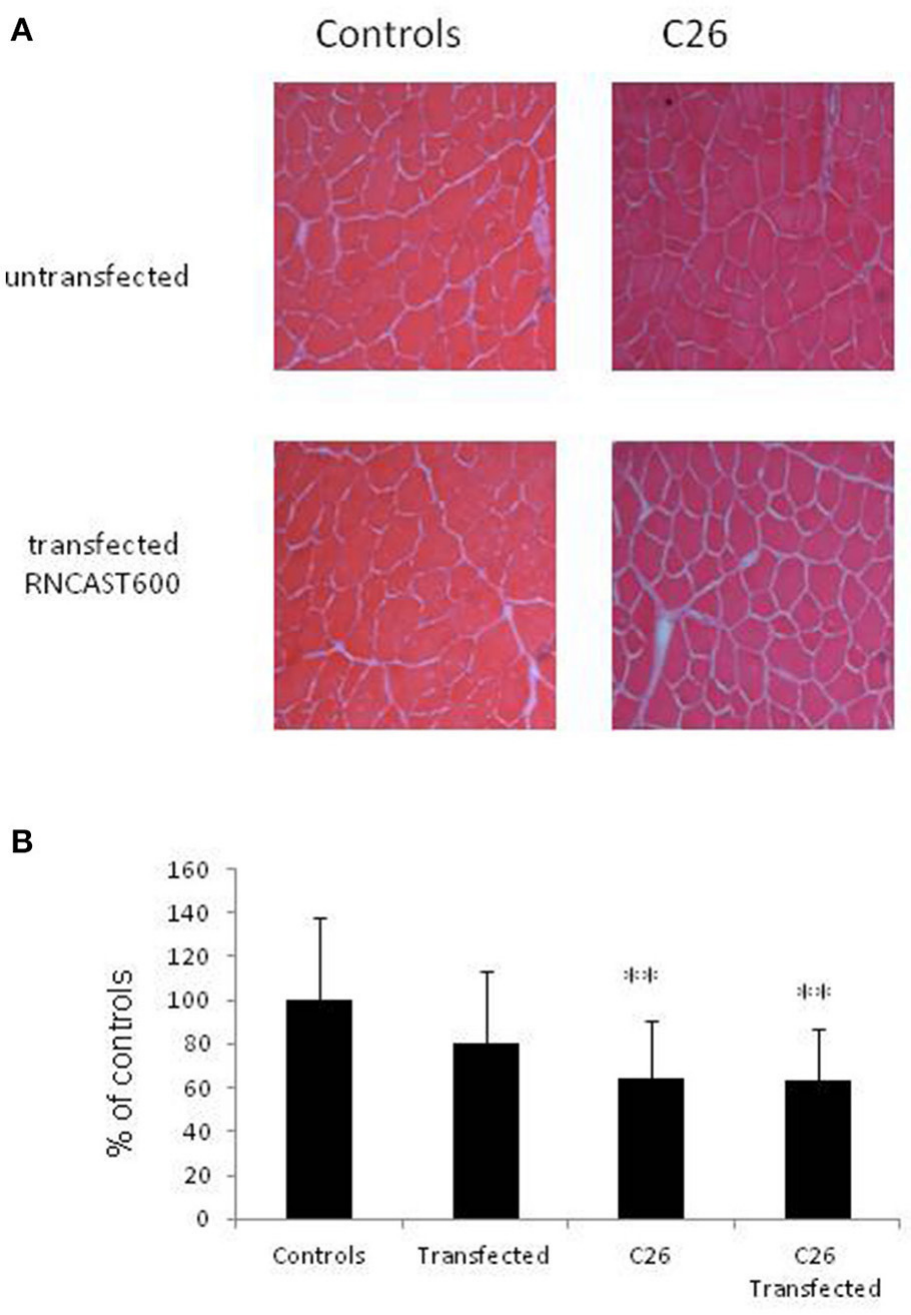
**Myofiber cross-sectional area. (A)** Representative histological pattern (hematoxylin and eosin staining) of transfected and untransfected muscles. **(B)** Morphometric analysis performed on hematoxylin and eosin stained sections of tibialis muscle derived from controls and C26 tumor-bearing mice untransfected or transfected with RNCAST600. Data (mean ± *SD, n* = 8) are expressed as percentages of controls. Significance of the differences (ANOVA): ^**^p < 0.01 vs. controls.

The use of systemic inhibitors is limited by pharmacokinetics, biological effective dose and potential toxicity. For this reason, as a second strategy to specifically target calpain activity locally in the skeletal muscle, a gene approach aimed at hyperexpressing calpastatin was used. The left *tibialis anterior* muscle of mice bearing the C26 tumor was transfected with an expression vector encoding a calpastatin species containing, in addition to a single inhibitory unit, also the regulatory L-domain (RNCAST600; transfection efficiency: 30%). The transfected sequence, even if produced as a fusion protein with the YFP tag at the C-terminus, maintains the ability to inhibit calpain activity (Figure S3; De Tullio et al., 2009). Being muscle transfection localized unilaterally to the *tibialis anterior*, no effects on body weight were observed, as expected (Figure 4A). RNCAST600 overexpression, however, was also unable to interfere with both loss of muscle mass (Figure 4B, Figure S4; Table S2) and changes of myofiber cross sectional area (CSA; Figure 5, Figure S4). A tendency to reduce muscle mass that does not reach significance, could be observed in control mice receiving electroporation. Despite not significant, such reduction in size could suggest that the transfection procedure might have produced a systemic reaction, likely of inflammatory nature, that could slightly affect the whole muscle compartment and that could have been partially antagonized in tumor hosts. However, the observation that no differences were observed in terms of CSA (Figure 5B, Figure S4) supports the lack of effectiveness of RNCAST600 overexpression in preventing C26-induced muscle atrophy. RNCAST600 overexpression did not affect tumor mass (C26 = 355 ± 61 mg, C26-RNCAST600 = 326 ± 58 mg).

## Discussion

The results shown in the present study demonstrate that the expression of molecules pertaining to the calpain-dependent proteolytic pathway is altered in the muscle of tumor hosts, indicating an enhanced activity of this system and confirming previous reports (Costelli et al., 2005). Most importantly, however, inhibiting this proteolytic system does not contrast cancer-induced muscle wasting.

By all available evidence, the calpain system does not contribute significantly to bulk protein degradation, but only performs a limited proteolysis of substrates. Along this line, in normal conditions of calcium homeostasis calpains basically exert a processing rather than a degradative action aimed to induce functional modifications in the target proteins (Goll et al., 2003). Calpains directly recognize substrates, differently from the lysosomal and the proteasomal systems that need ubiquitylation or sequestration into autophagosomes to tag their substrates (Goll et al., 2003). It is now well-accepted, in this regard, that while calpains are required to initiate the degradation of myofibrillar proteins, they are not involved in the bulk degradation of sarcoplasmic or sarcolemmal proteins (Huang and Zhu, 2016). Calpains appear to sever thick and thin filaments from myofibrils, thereby permitting a subsequent step in which suitable protein substrates are allowed to gain access to the degradative machinery (proteasome and lysosomal-dependent systems: Reviewed in Huang and Zhu, 2016). The activity of such a physiologically operating pathway can be enhanced by homeostasis alterations. As an example, oxidative stress was shown to increase calpain expression in cultured myocytes (Dargelos et al., 2010) and to induce the degradation of myofibrillar proteins (Smuder et al., 2010). Of interest, oxidative stress was shown to occur in the skeletal muscle of cachectic tumor-bearing animals (Mastrocola et al., 2008; Assi et al., 2016).

The calpastatin construct (RNCAST600) used in the present study encodes a single inhibitory unit and a complete regulatory L-domain. Previous data show that YFP-tagged RNCAST600 retains its ability to inhibit calpain activity (Stifanese et al., 2008; De Tullio et al., 2009) and since it contains the L domain, it can bind calpain also in the inactive form (Melloni et al., 2006). In addition, we have here observed that 1 week after transfection RNCAST600 is still expressed, not degraded, and effectively inhibits calpain activity (Figure S1). The lack of effect of RNCAST600 in preventing cancer-induced muscle wasting could depend on a sort of “calpastatin resistance”. A loss of inhibitory efficiency, that allows calpain to be active even at high concentrations of calpastatin, was previously observed in those calpastatin forms containing also the regulatory region (XL-L and L-domains; De Tullio et al., 2014). Following [Ca^2+^]_i_ increase, calpastatin, that is normally localized in perinuclear aggregates, diffuses in the cytosol and interacts with active calpain. Such diffusion can be reversed, even in the presence of Ca^2+^, if exon 6-containing calpastatins are phosphorylated by PKA (De Tullio et al., 1999). This process ultimately leaves active calpain free to operate on its targets even in the presence of high amounts of inhibitor. The possibility that these regulations contribute to the lack of effect of calpastatin overexpression in the muscle of the C26 hosts cannot be discarded. Future challenges will be to explore whether the transfection of different forms of calpastatin (i.e., without L-domain or with an L-domain lacking exon 6) could affect cancer-induced muscle wasting.

In addition to myofibrillar protein degradation, calpain activation also positively modulates endoplasmic reticulum (ER) stress (Muruganandan and Cribb, 2006; Langou et al., 2010). Muscle wasting induced by cancer was enhanced by inhibiting both ER stress and unfolding protein response (UPR) (Bohnert et al., 2016). An intriguing speculation, in this regard, is that calpain inhibition could result in reduced myofilament release from sarcomeres, but also in ER stress and UPR down-regulation (Isaac et al., 2016), still shifting protein turnover toward degradation.

The results shown in the present study support the consolidating notion that modulating the activity of a single proteolytic system would not be an adequate strategy to defeat cachexia. Indeed, previous reports showed that proteasome specific inhibition was unable as well to counteract muscle wasting in animals bearing experimental tumors (Fermoselle et al., 2013; Chacon-Cabrera et al., 2014; Penna et al., 2016). This is not totally unexpected, taking into consideration that muscle protein homeostasis (proteostasis) results from the convergent regulation of protein synthesis and degradation rates. These latter depend on the activity of the whole protein catabolic machinery, with the proteolytic pathways acting in concert, thereby providing substrates for *de novo* protein synthesis and energy production.

**Figure 6:**
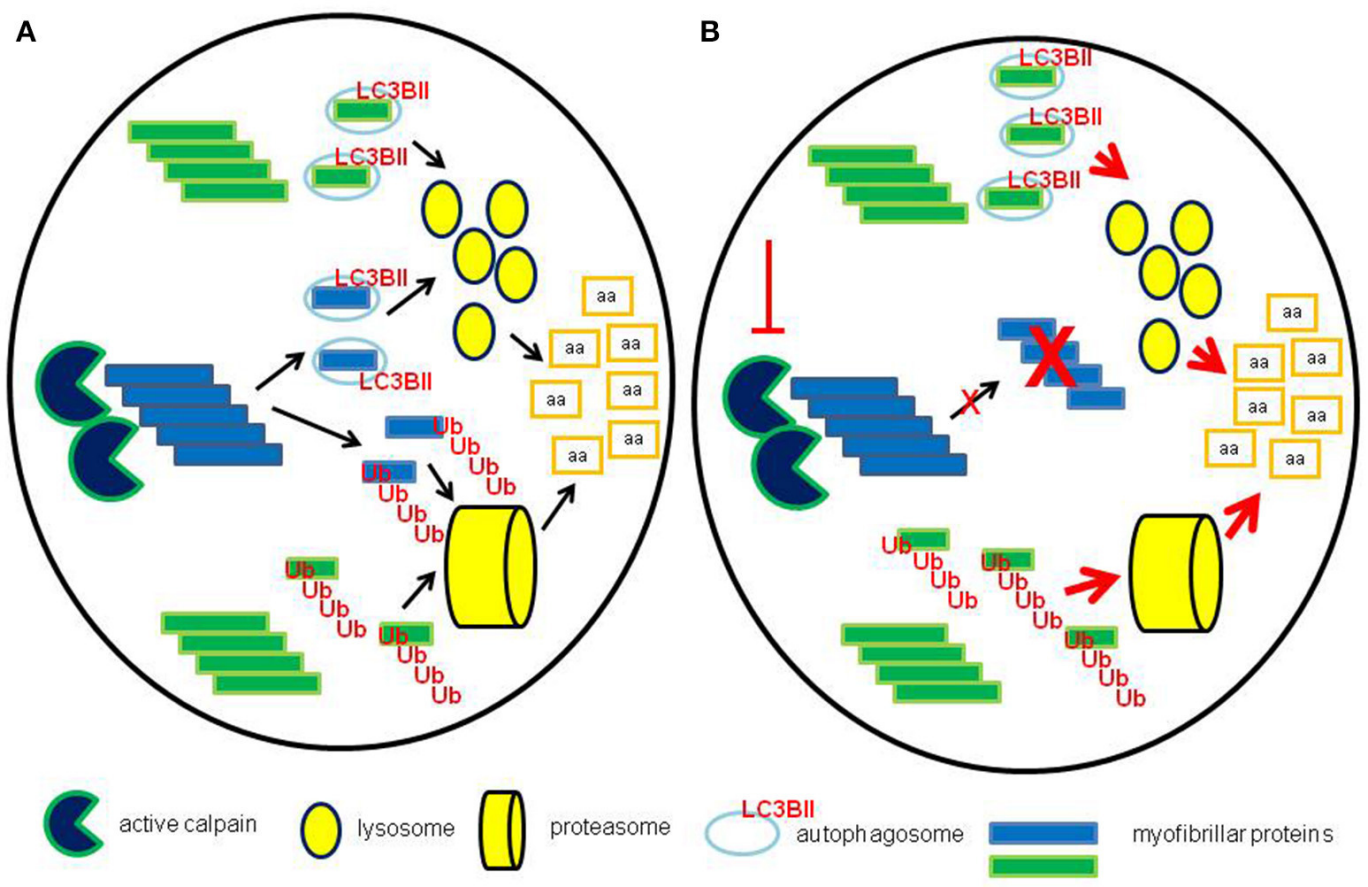
**Maintenance of proteostasis after calpain inhibition. (A)** In normal conditions calpains contribute, together with other systems such as caspases, to the degradation of myofibrillar proteins by severing thick and thin filaments from myofibrils, providing substrates for both proteasome and lysosomal-dependent systems. **(B)** When calpains are inhibited, pharmacologically or by genetic manipulation, the activity of both lysosomes and proteasomes is likely enhanced, in the attempt to maintain the physiological rates of protein degradation.

The interplay among the different proteolytic systems likely allows the myofiber to preserve proteostasis. If a single proteolytic pathway is failing, the others might well-increase their activity, aiming to maintain the fractional degradation rates at physiological levels (Figure 6). As an example, inhibition of the ubiquitin-proteasome system led to increased autophagy, while proteasome-dependent proteolysis was activated when autophagy was blocked (Yamaguchi et al., 2012). Similarly, calpain inhibition induced autophagy by reducing the levels of the Atg12-Atg5 conjugate and/or by degrading beclin-1 (Yamaguchi et al., 2012). The exogenous inhibition of a single proteolytic pathway could be associated with inefficient disposal of altered proteins/organelles by the other systems, overcoming the ability to maintain myofiber homeostasis, thus contributing to the wasting phenotype. Such a hypothesis appears particularly intriguing in cancer cachexia, where, in face of enhanced bulk proteolysis, the autophagic-lysosomal system is not able to efficiently get rid of accumulated proteins (Penna et al., 2013; Pigna et al., 2016).

On the whole, accumulating evidence indicate that most of the components of the muscle proteolytic machinery (calpains, proteasomes, lysosomes) are overexpressed in cancer cachexia, while systems involved in the endogenous control of protein turnover, such as calpastatin and deubiquitylating enzymes (Goll et al., 2003; Wing, 2016) are down-regulated. These changes are often considered as controlling events, however they might well-reflect an adaptive response to sustained hypercatabolic stimuli. The above reported observation that calpains appear to act upstream of both proteasome and lysosomal proteolysis supports the existence of a hierarchy in the hypercatabolic response in muscle wasting. In this regard, (upstream) therapeutic strategies should be focused on the real trigger(s) and on controlling events rather than on the single (downstream) proteolytic pathways.

## Author contributions

Conceived and designed the experiments: PC, MM, and RD; Performed the experiments: FPin, VM, and FPenna; Analyzed the data: RD and PC; Contributed reagents/materials/analysis tools: RD; Wrote the paper: FB and PC.

### Conflict of interest statement

The authors declare that the research was conducted in the absence of any commercial or financial relationships that could be construed as a potential conflict of interest.
