# Supplementary material for: Interference with Ca2+-Dependent Proteolysis Does Not Alter the Course of Muscle Wasting in Experimental Cancer Cachexia
Source: Front Physiol. 2017 Apr 19;8:213. doi: 10.3389/fphys.2017.00213 (PMC5395607; doi:10.3389/fphys.2017.00213)
Supplement: Supplementary file 1 [file Table1.PDF]

	b.w.	GSN	soleus	heart
**C**	203±11	582±42	38.7±1.2	504±63
**dantrolene**	208±16	553±67	34.8±2.3	499±46
**AH-130**	144±19*$	389±43*$	28.6±3.0*$	428±51*$
**AH-130 dantrolene**	141±15*$	361±55*$	26.3±2.7*$	393±50*$

Table S1. Effects of dantrolene treatment in AH-130 tumor-bearing rats.
Body weight (b.w.), gastrocnemius (GSN), soleus and heart weight in controls and AH-130 bearing rats, treated or not with dantrolene. In the AH-130 groups, b.w. is exclusive of tumor mass. Data (mean ± SD) are expressed as g (b.w.) or as mg/100 g initial body weight (muscles). Significance of the differences: * p<0,05 *vs* controls and $ p<0.05 *vs* dantrolene.

	C	RNCAST600	C26	C26 RNCAST600
**GSN**	664 ± 15	553 ± 30 *	404 ± 45 *** $$$	444 ± 54 *** $$$
**tibialis untransfected**	217± 19	194 ± 19	136 ± 28 *** $$$	144 ± 32 *** $$$
**tibialis transfected**	212 ± 13	182 ± 10	132 ± 24 *** $$$	157 ± 19 *** $$$

Table S2. Effects of RNCAST600 transfection on muscle weight in controls and C26 tumor-bearing mice.
Gastrocnemius (GSN) and tibialis weight (mean±SD) expressed as mg/100 g initial body weight. Significance of the differences: *p<0,05; ***p<0,001 *vs* controls and $$$ p<0,001 *vs* RNCAST600.
