# Supplementary figures and images for: Interference with Ca2+-Dependent Proteolysis Does Not Alter the Course of Muscle Wasting in Experimental Cancer Cachexia
Source: Front Physiol. 2017 Apr 19;8:213. doi: 10.3389/fphys.2017.00213 (PMC5395607; doi:10.3389/fphys.2017.00213)

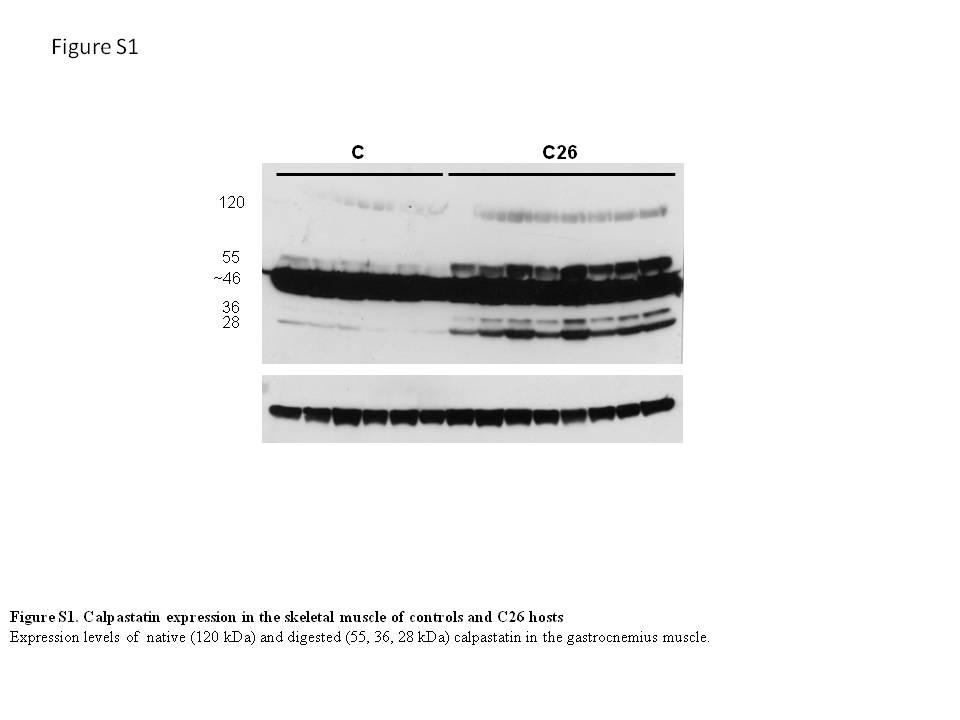

Supplement: Supplementary file 2 [file Image1.JPEG]

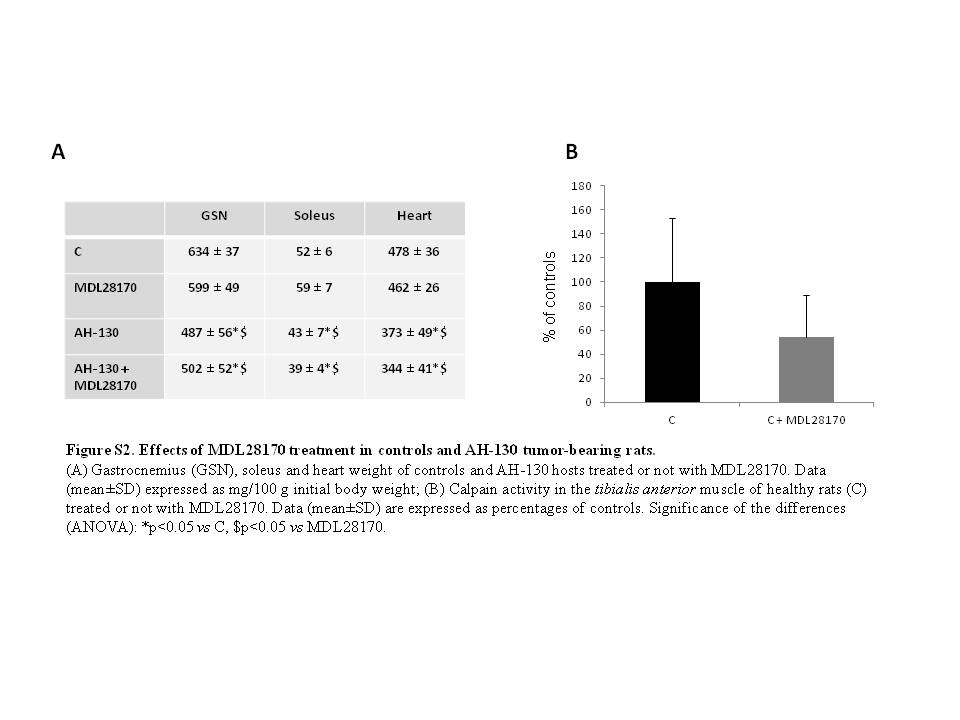

Supplement: Supplementary file 3 [file Image2.JPEG]

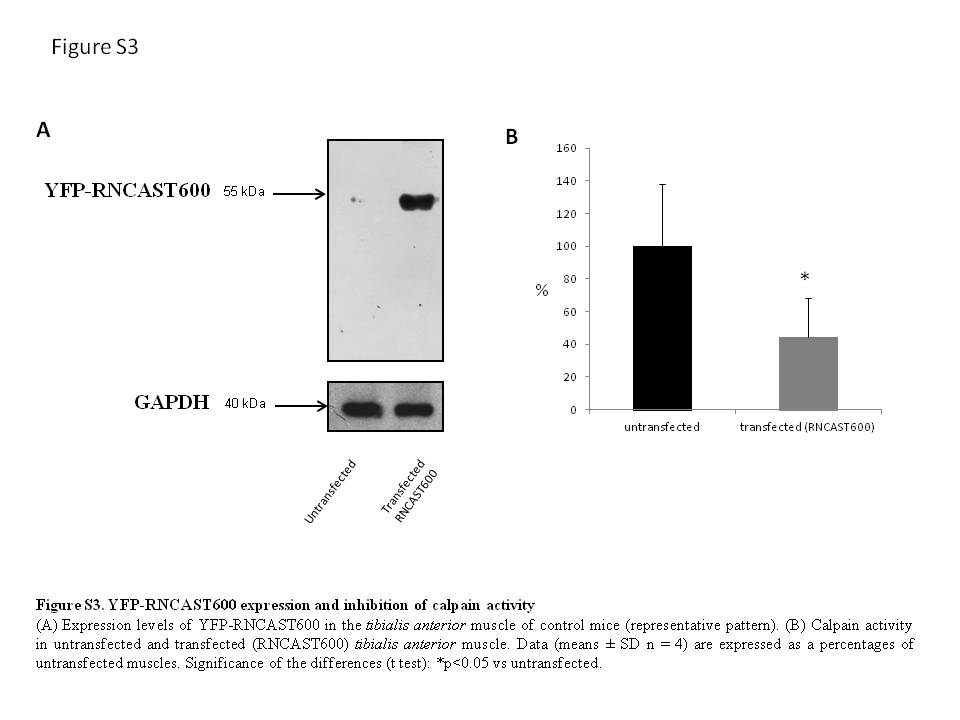

Supplement: Supplementary file 4 [file Image3.JPEG]

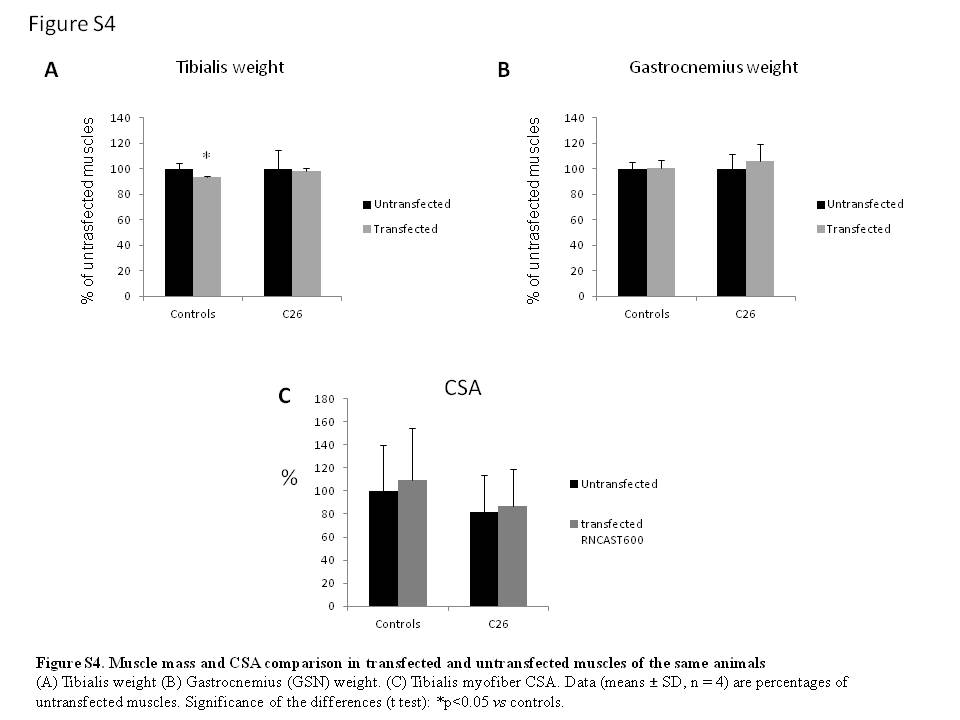

Supplement: Supplementary file 5 [file Image4.jpg]
